# Veterinary and Equine Science Students’ Interpretation of Horse Behaviour

**DOI:** 10.3390/ani7080063

**Published:** 2017-08-15

**Authors:** Gabriella Gronqvist, Chris Rogers, Erica Gee, Audrey Martinez, Charlotte Bolwell

**Affiliations:** 1 Massey Equine, Institute of Veterinary, Animal and Biomedical Sciences, Massey University, Private Bag 11-222, Palmerston North 4442, New Zealand; g.gronqvist@massey.ac.nz (G.G.); c.w.rogers@massey.ac.nz (C.R.); e.k.gee@massey.ac.nz (E.G.); 2 École Nationale Vétérinaire de Toulouse 23 Chemin des Capelles, BP 87614, 31076 Toulouse CEDEX 3, France; a.martinez_14@envt.fr

**Keywords:** horse behaviour, horse welfare, qualitative analysis, expressive behaviour

## Abstract

**Simple summary:**

We assessed first-year veterinary science and veterinary technology and undergraduate equine science students interpretation of expressive horse behaviours. Previous experience with horses appeared to influence the students’ perception of the horses’ behaviour. Qualitative assessments of horse behaviour may be a useful tool for assessing students’ knowledge of horse behaviour.

**Abstract:**

Many veterinary and undergraduate equine science students have little previous horse handling experience and a poor understanding of horse behaviour; yet horses are one of the most unsafe animals with which veterinary students must work. It is essential for veterinary and equine students to learn how to interpret horse behaviour in order to understand demeanour and levels of arousal, and to optimise their own safety and the horses’ welfare. The study utilised a qualitative research approach to investigate veterinary science and veterinary technology and undergraduate equine science students’ interpretation of expressive behaviours shown by horses. The students (N = 127) were shown six short video clips and asked to select the most applicable terms, from a pre-determined list, to describe the behavioural expression of each individual horse. A wide variation of terms were selected by students and in some situations of distress, or situations that may be dangerous or lead to compromised welfare, apparently contradictory terms were also selected (happy or playful) by students with less experience with horses. Future studies should consider the use of Qualitative Behavioural Analysis (QBA) and free-choice profiling to investigate the range of terms used by students to describe the expressive demeanour and arousal levels of horses.

## 1. Introduction

Horses are one of the most dangerous animals that veterinary students have to learn how to handle correctly [1], in part due the innate flight response of this species [2]. It has been proposed that many of the accidents involving horses can be attributed to breakdowns in human-horse communication [3]. Thompson et al. [4] suggested that people with a poor understanding of horse behaviour may be at an increased risk of injury, as their ability to anticipate unwanted, yet natural, horse behaviours may be lacking. Veterinary students often lack previous horse experience and an understanding of horse behaviour, as many now come from an urban background [5,6,7,8]. In New Zealand, it has been reported that 60% of veterinary students were from cities and only 18% were from rural areas [6].

A cross-sectional survey in Australia reported that most mixed animal (59%) and large animal (65%) veterinarians had suffered severe acute injuries or chronic musculoskeletal injuries at work [9,10,11]. Studies in other countries have reported similar statistics, suggesting that such findings are not unique to Australian veterinarians [12,13]. An improved ability of veterinarians to assess behavioural cues from the horse, including changes in its arousal and affective state, could improve human-horse communication and potentially prevent some of these accidents [3,14].

Accurately assessing horse arousal and affective states would also benefit the horses in regard to their welfare. The affective state of an animal is a reflection of the animal’s welfare, according to The Five Domains model [15]. The Five Domains model was initially developed to evaluate welfare compromise in animals used in research, teaching, and testing [16]. This model provides a method for recognising compromise in the four physical domains (nutrition, environment, health, and behaviour) and in one mental domain, which is the animal’s affective experiences and reflects the animal’s overall welfare state [15]. In order to maximise welfare, it is important for veterinary students to learn how to assess the horse’s affective state in addition to the physical states [15,17].

Key features such as previous experience, training, and familiarity with the animal can influence what a person working with horses brings to a situation [18], which can either compromise or enhance the welfare status of the horse [19]. Whilst we might not expect veterinary students to become ethologists, it is important that they have a good understanding of horse behaviour and animal welfare science to ensure the safety of the student, so instances of poor welfare can be recognised [17]. However, there are currently no studies on the baseline level of student awareness or knowledge of animal behaviour on entry to their undergraduate equine science or veterinary courses, and the impact prior experience with horses and other animals may have on this. 

Whole animal profiling is a subjective or qualitative technique commonly used in the assessment of animals’ demeanour and behaviour (body language) [20,21]. Such methods rely on a human observer’s ability to note apparent details of an animal’s expressive behaviour, using “whole animal” descriptors such as playful, content, calm, or frustrated [22,23,24]. As part of this qualitative assessment of behaviour, predefined terms can be provided to the observers who are asked to score the strength of the expressive behaviour on a scale provided [21]. The current study utilised a qualitative analysis to investigate students’ interpretation of expressive horse behaviour in various contexts and to determine the influence of previous experience with horses on their interpretation of the behaviours shown.

## 2. Materials and Methods 

The study was conducted using first-year veterinary science or veterinary technology students and students enrolled in undergraduate equine science courses at Massey University. The students (N = 127) were informed that the aim of the project was to investigate the words used by students to describe horse behaviour. Written consent was obtained from each student and the project was evaluated as being a low risk project by the Massey University Human Ethics committee (No. 4000015518).

The study was based on short video recordings, rather than live animals, of everyday situations that were not set-up or specifically created for the purposes of this study. The testing was conducted at the start of the first semester, before the students received lectures or practical training on horse behaviour. Students were briefed verbally and in writing on the methodology of the study, and the testing procedures were explained in detail. The students provided demographic information including: gender (“male”, “female”, or “other”), age category “<20 years”, “21–25 years”, “25–30 years”, or “>30 years”) and previous horse experience (“None—never interacted with a horse prior to the start of this paper”, “Little—interacted with or ridden horses a few times under supervision”, “Some—interacted with or ridden horses regularly under supervision”, “Experienced—interacted with or ridden horses regularly unsupervised” or “Very Experienced—competitive rider or worked in the horse industry”). 

The protocol consisted of a test video, which was played prior to the start of the testing and 6 short video clips (approximately 10 s long) (Table 1). After each video, the students had 30 s to score the horse behaviour using 15 pre-selected fixed terms [25] adapted from Minero et al. [26] (Table 2). For this study, the term “responsive” was changed to “alert” to avoid confusion with a “responsive horse”, which is used regularly in reference to a horse’s response to a rider’s aids.

Data collection sheets were provided to the students, and each page contained the 15 pre-selected terms, listed in the same order for each video, and a scale between 1 (Weak) and 5 (Strong). The students were asked to circle the number that represented the strength of the behaviour they perceived to best describe the horses in the individual videos. The students could choose more than one term for each video, and students were not made to select a score for every term for each video. 

### Statistical Analysis

Data were entered into Microsoft Excel and summarised using Pivot tables to describe the demographic variables. The number and percentage of students selecting each term for each video was calculated. For each video, the median score and interquartile range (IQR) for each term was calculated to describe the strength of the selected term. Multiple Correspondence Analysis (MCA), with the joint option in Stata, was used to visualise the relationship between the students’ previous experience and the terms selected (in binary form presence or absence of behaviour) for each video. MCA is a descriptive technique used to visualise binary and categorical variables of interest (student experience) as points on a two-dimensional plot to describe how strongly and in which way the variables are related or cluster together [27]. The variables were represented on the plot by a symbol and one for terms that were selected by students and zero for terms that were not selected. The variables that explain each axis (or dimension) can be determined by their position on the graph. The points that are clustered together are considered similar to each other, whereas those plotted furthest apart are rarely associated with each other. The centre of the plot represents the average profile and is said to be homogeneous, so points in this position are very similar to the average profile [27]. All analyses were conducted in Stata version 14.1

## 3. Results

Data were collected from 127 students that agreed to take part in the study. Most students were female, aged <20 years old, and enrolled in the veterinary science or technology programme (Table 3). Just under half of the students rated themselves as being either Experienced or Very Experienced, with 10% of students indicating they had no previous experience with horses (Table 3).

The number of students selecting each term and the median score for each selected term for each video is shown in Table 4. The students selected 13/15 terms for Video 1, of which most students strongly perceived the horse as being Alert (80%) and Curious (84%), and 81% of students scored the horse as moderately Friendly (Table 4). Just over half of students (54%) gave moderate scores for At Ease, and 30% of students gave moderate scores for Happy. One student with no experience with horses selected strongly Fearful, and two students with little or some experience selected weakly Fearful, whilst five students with little or some experience with horses, and one Experienced student selected Uncomfortable. Withdrawn and Agitated were selected by five and two students, respectively, whose experience with horses was little or none.

Video 2 was characterised by six terms and over 80% of students scored the horse as strongly Agitated, Distressed, and Uncomfortable; just under 80% of students strongly scored the horse as Anxious and Fearful (Table 4). Moderately Playful was selected by one student, and weakly Happy and At Ease were selected by one student each for Video 2. Of these 3 students, one had Little Experience, one was Experienced, and one was Very Experienced with horses. Multiple correspondence analysis for the terms for Videos 1 and 2 did not show any clustering with student experience (data not shown). 

**Figure 1:**
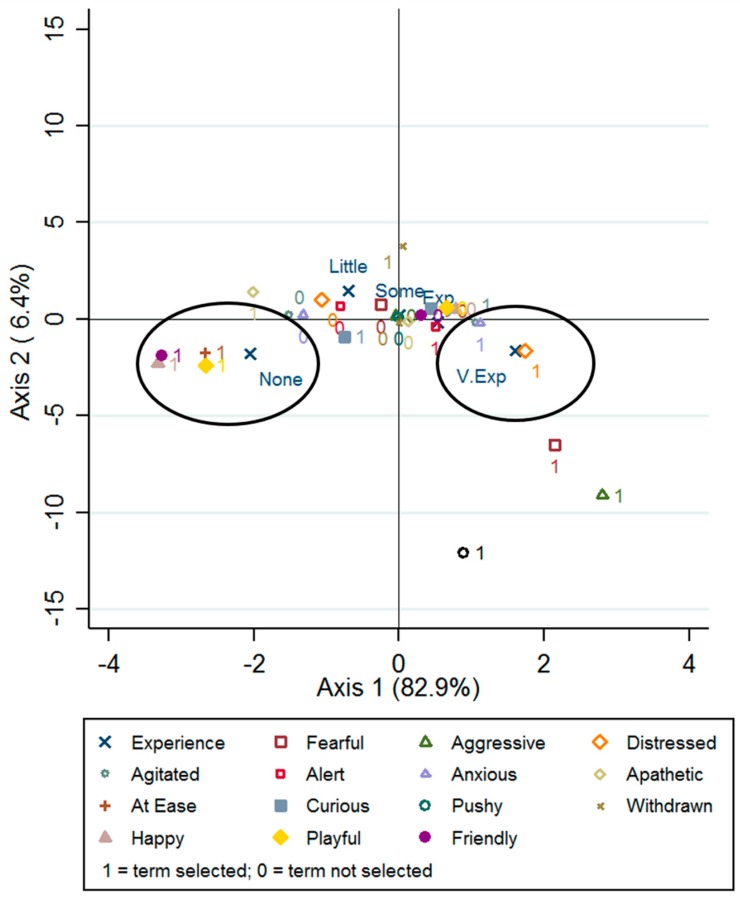
Multiple correspondence analysis showing the terms selected by first-year veterinary science or veterinary technology and undergraduate equine science students to describe the horse’s behaviour shown in Video 3, and the level of the students’ experience with horses. 1 = term selected at any strength; 0 = term not selected. ‘Experience = None—never interacted with a horse prior to the start of this paper’, ‘Little—interacted with or ridden horses a few times under supervision’, ‘Some—interacted with or ridden horses regularly under supervision’, ‘Experienced—interacted with or ridden horses regularly unsupervised’ or ‘Very Experienced—competitive rider or worked in the horse industry’. Black circles are used to indicate clusters in the data.

All terms were selected for Videos 3 and 5 (Table 4). For Video 3 over half of students perceived the horse to be strongly Agitated, Alert, Curious, and moderately Anxious, with 20%, 20%, and 19% of students perceiving the horse to be Moderately At Ease, Playful, and Happy, respectively. Of the students selecting moderately Happy, Playful, or At Ease, 67% (16/24), 68% (17/25) and 72% (18/25) had little or no previous experience with horses, respectively. Multiple Correspondence Analysis for Video 3 showed that Axis 1 (first dimension) was characterised by the terms Distressed, Agitated, Anxious, At Ease, Happy, Playful, and the previous experience of students (No Experience or Very Experienced) (Figure 1). The plot shows that students with no previous experience with horses clustered with the terms Friendly, Playful, Happy, and At Ease (Figure 1) in the same direction away from the average profile (in the centre of the plot). Very Experienced students clustered with the terms Distressed, Anxious, and Agitated, and this cluster was in an extreme opposite position to the other cluster. 

Most students selected that the horse in Video 4 was strongly At Ease or Apathetic, with 41% of students selecting moderately Withdrawn and 17% selecting Happy (Table 4). Some students selected Anxious or Uncomfortable, of which 81% (13/16) and 64% (9/14) of the students had little or no experience with horses. Two students with no previous experience with horses perceived the horse in Video 4 to be strongly Anxious, Distressed, and Uncomfortable, whilst four students with Little or Some Experience and one Experienced student rated the horse as Distressed. Five students with little or no experience and one Experienced student rated the horse as fearful. 

Most students (91%) perceived the horse in Video 5 to be Alert (highest score), with 61%, 57%, and 48% of students scoring the horse as moderately Anxious, Curious, and Agitated, respectively (Table 4). The horse in Video 5 was given weak scores for Happy, Friendly, At Ease, and Playful by 8%, 7%, 7%, and 4% of students, respectively, of which 6/10, 5/9, and 6/9 students had little or no experience with horses, respectively. 

Eight terms were selected for Video 6, with 94%, 87%, and 81% of students perceiving the horse to be Friendly, Happy, and At Ease (Table 4). Pushy was selected by four students, of whom three had Little Experience and one student was Very Experienced with horses, and Apathetic was selected by three students, of whom two had Little Experience and one was Experienced with horses. Multiple correspondence analysis for the terms for Videos 4, 5, and 6 did not show any strong clustering with student experience (data not shown). 

## 4. Discussion

This study aimed to assess first-year veterinary science or veterinary technology and undergraduate equine science students’ interpretation of expressive behaviour shown by horses. Across all videos, a range of terms were selected at varying strengths, and in some instances the percentage of students selecting a term was clustered with the students’ level of experience with horses. Whilst most students selected similar terms to describe the horse’s behaviour in a video, apparently contradictory terms were also selected for some videos. For example, terms describing negative affective states (Agitated, Anxious, Fearful, and Uncomfortable) were selected for the videos showing a horse resting with eyes half closed and the handler catching the horse from the paddock. Of concern was the apparent confusion of the expressive behaviour exhibited by the horse alone in an outside yard with positive affective states (Curious, Playful, Happy, and At Ease). 

Misinterpretation and failing to recognise behaviours such as anxiety may be an animal welfare concern. A horse in isolation is unable to carry out interactive behaviours with other horses and experiences constraints on its environment (unable to escape), which results in negative affective states such as loneliness, anxiety, fearfulness, and panic [15,28]. Such negative affective states ultimately result in a negative welfare status for the horse. The importance of veterinary and equine science students learning to identify negative affective states and situations that may be predictive of dangerous or aggressive behaviour, and the potential for welfare compromise, should not be underestimated. Humans have a large influence over the welfare status of horses, and their knowledge, skills, training, and familiarity with the animal can compromise or enhance the horses’ welfare status [18,19]. People working with horses need to be able to anticipate and identify problems, as well as ensuring good welfare is maintained [19]. Integrating animal welfare science in the veterinary and equine science curriculum would ensure students can use a more holistic approach to assessing horse behaviour and welfare states [17,29].

An inability of veterinary and equine students to assess horse behaviour could create breakdowns in human-horse communication and subsequently pose a safety risk for the students [3]. The most common injuries during horse handling practicals at an Australian Veterinary School were inflicted by horses stepping on ankles or feet and by bites or hind limb kicks [30]. Results from the Australian study suggest an inability of the students to swiftly and accurately assess the affective state of the horse and respond accordingly. The behaviour expressed by an individual animal is believed to, at least in part, be a reflection of the individual’s present arousal level and affective state [29,31]. Furthermore, it is worth noting that there is wide variation in the behaviour expressed and the reactivity of horses. Riley et al. [30] reported that inattention and inexperience were cited by students as the cause of 30% and 39% of horse-related accidents, respectively. Moreover, 30% of students believed that the accident occurred due to the horse being distressed or fearful [30]. An improved ability of veterinary students and professionals to recognise dangerous and threatening, as well as subtle, behavioural cues from the horse, including changes in its arousal and affective state, could help to prevent some of these accidents [3,8]. 

Previous research using qualitative methods has reported differences between experienced and inexperienced observers [32] and between different stakeholders (farmers vs. animal scientists vs. urban citizens) [33]. In the current study, the students’ interpretation of the horses’ behaviour in one video was clustered with the students’ level of experience with horses. Additionally, across all videos, there were terms selected that appeared to be contradictory to the terms selected by most students and the behaviour expressed in the video, which were often selected by students with less experience. These results suggest that it may be useful to identify ‘at risk’ students with less experience of horses who may benefit from additional learning activities before practical handling sessions, which have previously been shown to successfully increase student awareness and understanding of animal behaviour [34]. However, it is likely that further studies are required to build on the baseline data provided in this study, in order to investigate the influence of previous experience with horses on students’ interpretation of behaviour and safety around horses.

A study examining inter- and intra-observer reliability of experienced and inexperienced observers of behaviour described differences with experience in how the observers perceived the terms and descriptors [32]. Observers with less experience were reported to have less correlation amongst terms compared to observers experienced in assessing dairy cattle [32]. Although future studies are required to specifically investigate the reliability of student observers, the results from the current study support preceding research suggesting that the way in which the observers perceive and interpret the descriptors may be affected by their previous experience [32]. These findings require further exploration to better understand how much and what type of experience with horses is useful (for example, ridden versus husbandry skills) to potential veterinary and undergraduate equine science students. 

The expressive behaviours of the horse may have been the driver in the videos where no pattern was demonstrated with the selected terms and student experience. It is possible that in some situations animal behaviours may be easily identified with general knowledge (rather than specific experience with horses) or by applying a human emotional interpretation [32]. Such behaviours may be considered more obvious, rather than subtle, behaviours that people with very little experience with horses (or other animals) may be able to recognise. Mutual grooming between horses appeared to be a behaviour that students with little previous horse experience could recognise. Mutual grooming is a common behaviour seen in a large number of group living species [35]. Perhaps the ease with which students identified mutual grooming was linked to the fact that this behaviour is commonly seen in different social species. Whereas, it may not always be clear to unexperienced students when horses are demonstrating fearful or anxious behaviour; such behaviours may be subtler to students that have no familiarity with identifying the changing arousal levels associated with the natural flight response in horses [29]. The hypothesis that students’ recognition of subtle or more obvious behaviours may differ is of importance when teaching students to work safely around horses in order to minimise harm.

Additionally, a number of videos included a handler with the subject horse (Table 1) and it is possible that the handlers in the videos provided the less experienced students with clues as to the arousal level of the horses, resulting in some contextual bias [24]. For example, students may be directed towards certain terms in Video 1 based on the behaviour (posture, facial expressions, behaviour) of the handler when catching the horse. Similarly, knowing that a handler is administering something to the horse in Video 2 may have biased the students towards terms that reflect negative affective states and interpreted the horse’s behaviour as Uncomfortable or Anxious. The compounding factors of the handler or the context of the video may be one explanation for the lack of strong clustering with student experience of horses in most videos. 

The current study used a fixed set of terms and descriptions adapted from Minero et al. [26] to assess the welfare of donkeys. These terms were selected for use in the current study due a lack of published studies using fixed terms to investigate the welfare, or otherwise, of horses. Furthermore, a thorough consultation process with experts in donkey welfare and behaviour was used by Minero et al. [26] to develop the list of terms and their descriptions. However, during the application to videos of horses in the current study, it became clear that a number of the descriptions overlapped or included other terms in the descriptions, potentially causing confusion amongst students. Minero et al. [26] noted that linguistic barriers may lead to confusion in the interpretation of descriptors and bilingual dictionaries were utilised to reach consensus when characterising the terms. Due to this, it is possible that there was some level of ambiguity in the terms in relation to the behaviour observed, which may have contributed to the wide variation of terms selected by students, and a lack of apparent association with experience, for some videos. Additionally, it is possible that the terms selected were suitable for donkeys, but were not suitable for assessing expressive behaviour in horses. Since the current study was completed a set of pre-defined terms for using Qualitative Behavioural Analysis (QBA) to assess horse welfare have been developed through the AWIN (animal welfare indicators) welfare assessment protocol for horses [36]. More emphasis may need to be put towards choosing the correct terminology in future studies [32], with consideration of the new terms developed specifically for horses [36]. Alternatively, there may be some merit in utilising a QBA approach with free-choice profiling and Generalised Procrustes Analysis [23] to assess students’ language used to describe the behaviour of horses.

## 5. Conclusions

Overall, a wide variation of terms were selected by students to describe the horse’s behaviour shown in each of the videos. In some situations, the ability to recognise expressive horse behaviour was associated with students’ previous horse experience. Some situations of distress, or those that may lead to compromised welfare or potentially dangerous situations were poorly identified by some students, and the reasons for this requires further investigation. Further improvements can be made to the qualitative approach used in this study to provide a novel mechanism for evaluating the students’ interpretation of horse behaviour and arousal level.

## Figures and Tables

**Table 1:** Descriptions of the six videos used in a study to assess students’ interpretation of expressive horse behaviour.

Video	Description
1	The handler and horse walk towards each other in a paddock. The handler strokes the horse and attaches the lead rope whilst the horse stands still.
2	The handler holds the horse with a halter and lead rope. The handler moves a worming syringe towards the horse’s mouth while the horse backs away. When the syringe reaches the mouth, the horse pulls back and canters away.
3	The horse is in an outside yard on its own. The horse initially walks quickly and then trots around the perimeter of the yard. Horse is lifting and lowering its head to the ground as it moves around the yard.
4	The horse stands alone in an arena with a saddle and halter on. The horse is resting with eyes half closed.
5	The horse is loose in an indoor yard on its own. The horse is standing still with its head elevated and ears pointing forward. The horse then moves off at a brisk walk around the yard.
6	The horse is in a yard with several other horses. The horse is partaking in mutual grooming with one other horse.

**Table 2:** The pre-selected terms and descriptions used in a study to assess students’ interpretation of expressive horse behaviour, based on Minero et al. [26].

Term	Description
Aggressive	Behaving in an angry or rude way, fighting or attacking
Agitated	Restless, fidgety, worried or upset, excited, disturbed, troubled
Alert	Receptive, aware of the environment
Anxious	Worried/tense, troubled, apprehensive, distressed
Apathetic	Having or showing little or no emotion, indifferent
At ease	In a relaxed attitude or frame of mind
Curious	Eager to learn, inquisitive, wishing to investigate
Distressed	Much troubled, upset, afflicted, panicking
Fearful	Having fear, afraid, displaying a flight response, looking anxious, back up/away
Friendly	Not hostile, showing positive feelings toward another horse or person
Happy	Feeling, showing or expressing joy, pleased
Playful	Very active, happy, and wanting to have fun, mischievous
Pushy	Offensively assertive or forceful, bossy, dominant
Uncomfortable	Not comfortable, not relaxed
Withdrawn	Secluded or remote, shy, not searching for contact with others

**Table 3:** The number and percentage of first-year veterinary science or veterinary technology and undergraduate equine science students, by gender, age, and level of previous experience with horses, used in a study to assess students’ interpretation of expressive horse behaviour.

**Demographic Variables**	**Number**	**Percentage**
Gender		
Male	17	13
Female	109	87
Age		
<20	100	79
21–25	23	18
25–30	2	2
>30	2	2
Level of experience		
None	13	10
Little	45	35
Some	13	10
Experienced	30	24
Very Experienced	26	20
Course studied		
Veterinary science or technology	66	52
Undergraduate equine science	61	48

**Table 4:** Number of students that selected each pre-defined term and the median (interquartile range) score for each term, selected by first-year veterinary science or veterinary technology and undergraduate equine science students, for each of the 6 videos used in a study to assess students’ interpretation of expressive horse behaviours (- = term not selected by students).

Word	Video											
	1		2		3		4		5		6	
	N	Median	N	Median	N	Median	N	Median	N	Median	N	Median
Aggressive	-	-	14	2 (1–3)	2	3 (1–4)	-	-	1	2 (2–2)	-	-
Agitated	2	2 (1–3)	104	4 (3–5)	75	4 (3–4)	2	2 (1–3)	61	3 (2–4)	-	-
Alert	102	4 (3–4)	67	4 (3–5)	79	4 (3–4)	17	2 (2–3)	115	5 (4–5)	8	3 (2–4)
Anxious	16	2 (2–3)	100	4 (3–5)	69	3 (2–4)	16	2 (1–4)	78	3 (2–4)	-	-
Apathetic	6	3 (3–3)	-	-	8	4 (3–4)	89	4 (4–5)	3	4 (2–5)	3	3 (2–4)
At Ease	68	3 (3–4)	1	1 (1–1)	25	3 (3–4)	88	5 (4–5)	9	3 (2–3)	103	4 (3–5)
Curious	107	4 (3–4)	4	4 (2–5)	47	4 (3–4)	1	4 (4–4)	72	3 (3–4)	20	3 (2–4)
Distressed	-	-	110	4 (4–5)	48	3 (2–4)	7	3 (2–5)	41	2 (1–3)	-	-
Fearful	3	2 (1–4)	100	4 (4–5)	13	2 (2–3)	7	2 (1–3)	17	2 (1–3)	-	-
Friendly	103	3 (3–4)	-	-	11	2 (2–2)	8	3 (1–3)	9	2 (2–3)	119	5 (4–5)
Happy	38	3 (3–4)	1	1 (1–1)	24	3 (2–4)	21	3 (2–3)	10	2 (2–3)	110	4 (3–4)
Playful	8	3 (2–4)	1	3 (3–3)	25	3 (2–3)	-	-	5	2 (1–2)	67	4 (3–4)
Pushy	2	2 (1–3)	8	3 (2–3)	1	4 (4–4)	-	-	1	2 (2–2)	4	3 (2–4)
Uncomfortable	6	3 (2–4)	106	4 (4–5)	53	3 (2–4)	14	3 (1–5)	53	3 (2–3)	-	-
Withdrawn	5	2 (2–2)	13	3 (2–5)	6	3 (2–3)	52	4 (2–4)	7	3 (2–4)	-	-
